# Microstructure and Mechanical Property Evaluation of Dune Sand Reactive Powder Concrete Subjected to Hot Air Curing

**DOI:** 10.3390/ma15010041

**Published:** 2021-12-22

**Authors:** Sara Ahmed, Zin Mahaini, Farid Abed, Mohammad Abdul Mannan, Mufid Al-Samarai

**Affiliations:** 1 Civil Engineering Department, American University of Sharjah, Sharjah 26666, United Arab Emirates; g00049654@alumni.aus.edu (S.A.); g00063338@alumni.aus.edu (Z.M.); 2 Civil Engineering Department, Universiti Malaysia Sarawak, Kota Samarahan 94300, Sarawak, Malaysia; mannan@unimas.my; 3 Civil Engineering Department, University of Sharjah, Sharjah 27272, United Arab Emirates; samarai@sharjah.ac.ae

**Keywords:** RPC, UHPC, curing regimes, GGBS, hot air curing, SEM

## Abstract

The use of different sustainable materials in the manufacture of ultra-high-performance concrete (UHPC) is becoming increasingly common due to the unabating concerns over climate change and sustainability in the construction sector. Reactive powder concrete (RPC) is an UHPC in which traditional coarse aggregates are replaced by fine aggregates. The main purpose of this research is to produce RPC using dune sand and to study its microstructure and mechanical properties under different curing conditions of water curing and hot air curing. The effects of these factors are studied over a long-term period of 90 days. Quartz sand is completely replaced by a blend of crushed and dune sand, and cement is partially replaced by using binary blends of ground granulated blast furnace slag (GGBS) and fly ash (FA), which are used alongside silica fume (SF) to make a ternary supplementary binder system. Microstructural analysis is conducted using scanning electron microscopy (SEM), and engineering properties like compressive strength and flexural strength are studied to evaluate the performance of dune sand RPC. Overall, the results affirm that the production of UHPC is possible with the use of dune sand. The compressive strength of all mixes exceeded 120 MPa after 12 h only of hot air curing (HAC). The SEM results revealed the dense microstructure of RPC. However, goethite-like structures (corrosion products) were spotted at 90 days for all HAC specimens. Additionally, the use of FA accelerated the formation of such products as compared to GGBS. The effect of these products was insignificant from a mechanical point of view. However, additional research is required to determine their effect on the durability of RPC.

## 1. Introduction

Reactive powder concrete (RPC) is an innovative cement-based material with exceptional mechanical and durability properties. Richard and Cheyrezy explained the key principles for developing RPC, including the elimination of coarse aggregates, the reduction in water-binder ratio by using superplasticizers, and the incorporation of small-sized steel fibers [1]. In order to prepare RPC, very fine powders such as Portland cement, silica fume, and quartz powder are utilized. Granular packing of these powders is optimized to achieve maximum density. Thus, RPC can attain compressive strengths greater than 150 MPa, while still maintaining good ductility due to the inclusion of steel fibers.

However, several problems hinder the production of RPC on a large scale. It is well known that the manufacturing of RPC requires high dosages of cement and silica fume. These high amounts not only increase the production cost, but also have adverse effects on the environment, as the cement industry has a very high carbon footprint. Thus, replacing cement and silica fume with mineral admixtures like fly ash (FA) or ground granulated blast furnace slag can resolve this issue [2]. Other types of sustainable cementitious materials, such as alkali-activated materials [3] and glass powder [4], can also be used in the manufacturing of RPC. For instance, test results by Yazici et al. [5] showed that RPC subjected to standard curing and with high volumes of GGBFS or FA achieved compressive strengths exceeding 200 MPa. This was possible even with cement contents as low as 376 kg/m^3^ [6]. There was also a better bond between the matrix and the steel fibers when high levels of GGBFS were added [7]. Therefore, better flexural performance was reported for RPC samples containing GGBS as compared to their control counterparts. Edwin et al. [8] reported that using copper slag as a supplementary cementitious material in RPC had a small positive impact on its compressive strength, which can be increased by using finer copper slag. The copper slag also enhanced the flowability of the RPC because of its low water absorption.

On the other hand, Hasan et al. [9] reported a significant reduction in the mechanical strength of RPC containing supplementary cementitious materials, FA and metakaolin, under standard curing. The reduction was more pronounced when the volume of FA increased. This can be due to the low activity index of the cementitious materials. Similarly, Mo et al. [10] found out that incorporating high amounts of GGBFS helped in mitigating the autogenous shrinkage but led to smaller strength values.

Several researchers have also investigated the possibility of using recycled powder from various waste materials in the production of RPC [11,12]. Vigneshwari et al. [12] investigated the effect of replacing silica fume with rice husk ash (RHA) on the behavior of RPC. RHA replacement levels ranged between 10 and 50%, and the RPC specimens were subjected to both standard and steam curing. The experimental results revealed that the microstructure of the concrete was enhanced with the addition of RHA, which led to an increase in the compressive, split tensile, and flexural strengths. Nonetheless, RPC samples with high RHA content showed superior durability. Likewise, an investigation was carried out to understand the behavior of RPC made from the recycled powder of construction and demolition wastes. A series of tests were conducted to measure the RPC performance for different replacement levels. It was concluded that replacing either silica fume or cement with recycled powder lowered the strength of the RPC. However, partial or complete replacement of silica fume with recycled powder still resulted in acceptable strength values, but in the case of cement, a replacement level of 18% was recommended [11].

Furthermore, the limited availability of quartz and its high cost necessitates the need for cheaper and more abundant materials. Some studies in the literature have proposed titanium slag [13] and glass powder [14] as feasible alternatives to crushed quartz. Titanium slag aggregates are a product of metallurgical enrichment of vanadium titanium magnetite which are produced in large amounts every year in China. The use of titanium slag in concrete, specifically in RPC, can reduce its negative effects on the environment [13]. Waste glass powder on the other hand in [14] was obtained from waste glass shards material which were grained to micron meter size and have properties similar to those of quartz powder [12]. Rong et al. [15] found out that replacing quartz sand with river sand reduced the compressive strength in the range of 9–12%, but the cost was also reduced by 10%. Likewise, Zhang et al. [16] investigated the effect of replacing quartz sand with natural river sand. The river sand had a much larger maximal diameter, 3 mm, than that of the ultra-fine quartz sand, 600 µm. Nevertheless, this green RPC, developed with slag and FA, was able to reach a compressive strength of 141.1 MPa under standard curing. Another investigation noted that using natural sand in place of crushed quartz can result in a denser microstructure, but lower compressive strengths. This is because quartz possesses better properties than natural sand, i.e., higher SiO_2_ proportions, greater hardness, and fewer impurities. However, the flexural strength of the quartz mixtures was somewhat similar to that of natural sand, because of the larger particle size of natural sand [17].

The use of ternary cementitious blends has also been studied in RPC and is desired in concrete in general for a number of reasons. Ternary blends can be used to enhance concrete properties, lower concrete production costs, and lower the embodied energy and carbon footprint of concrete [18]. Several studies have shown that the use of ternary blends of SF, GGBS, and FA perform better as compared to binary blends of either SF-GGBS or SF-FA when used alongside cement [16,19]. However, in a study conducted by Yazici et al. [5], it was shown that the use of SF-GGBS-FA only performed better compared to the binary blend of SF-FA and not SF-GGBS. Therefore, additional research is required to confirm this finding and also to investigate the types and doses of ternary blends which enhance the properties of the concrete. Bleszynski et al. [20] studied the effect of ternary blends (cement, SF, and GGBS) on concrete durability, and it was shown that ternary blends had higher resistance to chloride ingress as compared to cement only or mixes with single supplementary cementitious material (SCM).

Thus, green construction materials can be utilized to develop an eco-friendly RPC. In the scope of this study, a novel type of RPC was developed using local materials. The experimental program was designed to assess the influence of replacing 30% of cement with GGBS, using ternary blends in the production of RPC, and substituting quartz powder with crushed and dune sand. The fine aggregates used in this study had larger sizes (150 µm–1.18 mm) than those usually used in RPC (150 µm–600 µm). The behavior of the RPC was studied in terms of compressive and flexural strengths under varying curing conditions, and the microstructure of the RPC was examined using a scanning electron microscope (SEM).

## 2. Experimental Program

### 2.1. Characterization of Constituent Materials

**Figure 1:**
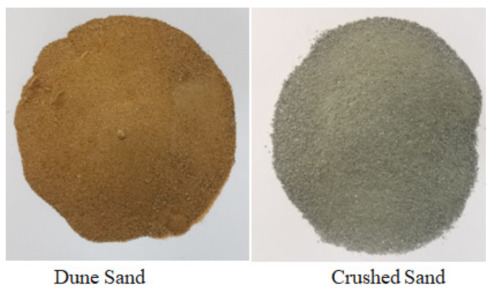
Fine aggregates.

**Figure 2:**
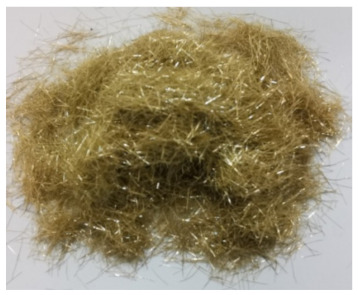
Steel fibers.

All the mixes were prepared using ordinary Portland cement (OPC) (CEM I 42.5) of specific gravity 3.14, silica fume (SF) of specific gravity 2.2, and a commercially available polycarboxylate-based high-range water reducing admixture. The OPC used was in compliance with BS EN 197-1:2011 [21] and had a Blaine fineness of 318 m^2^/kg. The ground granulated blast furnace slag (GGBS) and fly ash (FA) used to replace cement had a 417 m^2^/kg and 474.3 m^2^/kg Blaine fineness and confirmed to EN 15167-1:006 and IS 1727 [22], respectively. The chemical and physical properties of the binder materials are presented in Table 1 as per the manufacturer test certificate. The fine aggregates used were locally available in the UAE and included crushed and dune sand with specific gravities of 2.57 and 2.58, respectively. The crushed sand was supplied by Seven Star Trading in Dubai, UAE and was sieved to have particle sizes ranging from 150 μm to 1.18 mm. Brass-coated steel fibers 13 mm in length and 0.16 mm in diameter were used. The fibers had an aspect ratio of 87 and a tensile strength of 3000 MPa. Figure 1 and Figure 2 demonstrate the fine aggregates and steel fibers used in the study.

### 2.2. Mix Proportions

As previously indicated, RPC is mainly composed of high amounts of cement and SF (800–1100 kg/m^3^ and 150–300 kg/m^3^). The cement content in this study was reduced to 571 kg/m^3^ and RPC was produced using indigenous materials. Table 2 provides a summary of the mix designs of RPC. As shown in Table 2, abbreviations were used for mixes according to GGBS and/or FA content. GGBS and FA were denoted by GB and FA. The ratios by cement weight of the GGBS and FA were also given in the abbreviations. For example, GB15FA15 refers to the mix containing 15% GGBS and 15% FA, by cement weight. Moreover, CNTRL refers to the control mix which was prepared using cement and SF only. Note that the crushed sand and dune sand were used at a ratio of 1.38. All mixes had a constant water-to-binder (w:b) ratio, aggregate-to-binder (a:b) ratio, and steel fiber content of 0.17, 1.0, and 1.8% (by volume), respectively.

### 2.3. Mixing and Sample Preparation

In most of the studies, RPC is produced using three-stage mixing which involves mixing of all dry ingredients followed by the addition of water and superplasticizer. In [23], however, four stage mixing showed better flowability and compressive strength results compared to three stage mixing. Thus, in this study, four stage mixing method was adopted and the mixes were produced using a Hobart mixer. The mixing method involved the following sequence:

- Dry mixing of all binder materials at low speed (140 rpm) for 2 min;
- Addition of 80% of total water with 100% SP dosage and mixing at low-medium speed (140/185 rpm) for 3 min;
- Addition of fine aggregates and mixing at low-medium speed (140/185 rpm) for 4 min;
- Addition of remaining water and mixing at low-medium speed (140/185 rpm) until a fresh paste is formed (note: time for fresh paste varied based on binder materials used);
- Addition of steel fibers and mixing at low speed (140 rpm) for 3 to 6 min.

The mixing time for the mixes ranged from nearly 15 to 20 min depending on the binder material used. The control mix had a mixing time of 15 min, while the incorporation of GGBS and FA increased the mixing time to 18–20 min. The mixes were cast in molds and were compacted using a mechanical vibration table for approximately 1 min. After 24 h, the samples were removed from the molds and subjected to different curing conditions.

### 2.4. Curing Conditions

The mixes investigated in this study were cured at three different conditions, including:

- Water curing (WC) at 20 °C;
- Hot air curing (HAC) at 100 °C;
- Hot air curing at 100 °C + ambient.

For the third curing condition, the samples were kept in the oven for a duration of 24 h and then removed and left in ambient condition until their testing event. Note that for the HAC condition, after demolding the samples after 24 h, the specimens were left for an additional 24 h in ambient condition to ensure that the mix was completely dry before exposing to HAC (see Section 3.2). Therefore, the samples were put in the oven after approximately 48 h of their casting time.

### 2.5. Tests Conducted

#### 2.5.1. Workability

**Figure 3:**
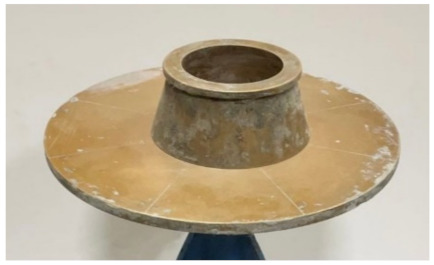
Mortar flow test apparatus.

The workability was evaluated using a flow of mortar test, which was performed as per ASTM C1437-20 [24]. The flow of mortar test apparatus is demonstrated in Figure 3. The diameter was measured along the two perpendicular lines scribed at the top of the table, and the flow was recorded as the average of two readings.

#### 2.5.2. Mechanical Tests

The compressive strength was performed as per ASTM C 109-20 [25] at a loading rate of 0.35 MPa/s on 50 × 50 × 50 mm specimens and tested at 3, 7, 14, 21, 28, 56, and 90 days for both water- and hot air-cured specimens. The HAC specimens were left in the oven for a duration of 24 h and were cured in ambient condition until their testing event (3, 7, 14, 21, 28, 56, and 90 days). Another group of mixes were cured in the oven for 48 h and the strength was monitored over a 12 h period to have an indication of the strength development of the specimens cured under HAC.

The flexural strength was performed as per ASTM C 293 [26] at a loading rate of 0.9 MPa/min on 40 × 40 × 160 mm prismatic specimens. The specimens were loaded from their mid-span, and the clear distance between the simple supports was 140 mm. The flexural specimens were cured in both WC and HAC and tested at 7, 28, and 90 days, respectively. A summary for the mechanical tests conducted, curing conditions employed, and testing events are shown in Table 3.

#### 2.5.3. Scanning Electron Microscopy

The microstructure of the RPC mixes was investigated using the SEM as per ASTM C1723 [27]. The accelerating voltage was 20 kV. The samples were prepared by taking small pieces of concrete from prismatic specimens which were later coated with gold to enhance the quality of the image. Secondary electron (SE) imaging was preferred to obtain the morphology. Backscattered electron (BSE) was also used to study the fiber-to-matrix bond in the mixes.

## 3. Results and Discussion

### 3.1. Workability

The flow for the CNTRL, GB30, and GB15FA15 mixes was 170, 185, and 185 mm, respectively. The use of GGBS and FA slightly increased the workability despite the increase in mixing time. This behavior may be attributed to the low water demand and the dense surface of the GGBS and FA particles, which only allow few water molecules to get adsorbed, thereby improving the workability at a fixed w:b ratio. This finding is in agreement with previous works [9].

### 3.2. Compressive Strength

**Figure 4:**
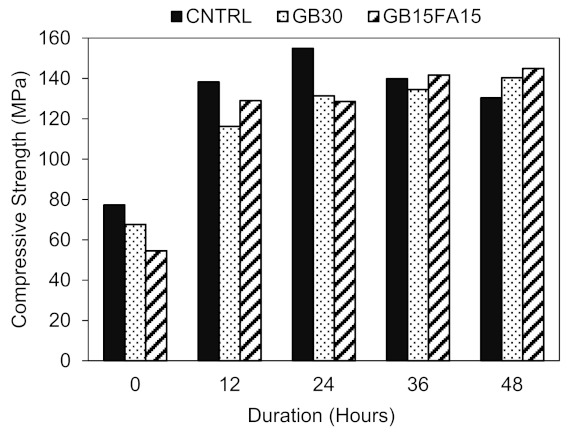
12 h HAC compressive strength development.

**Figure 5:**
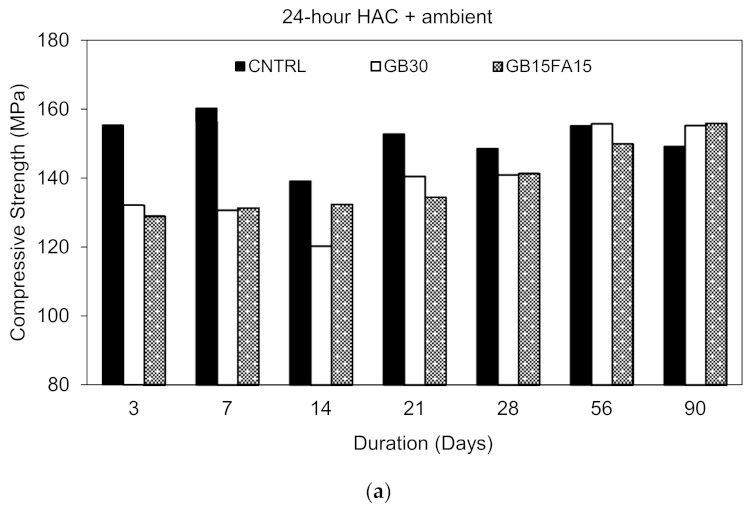

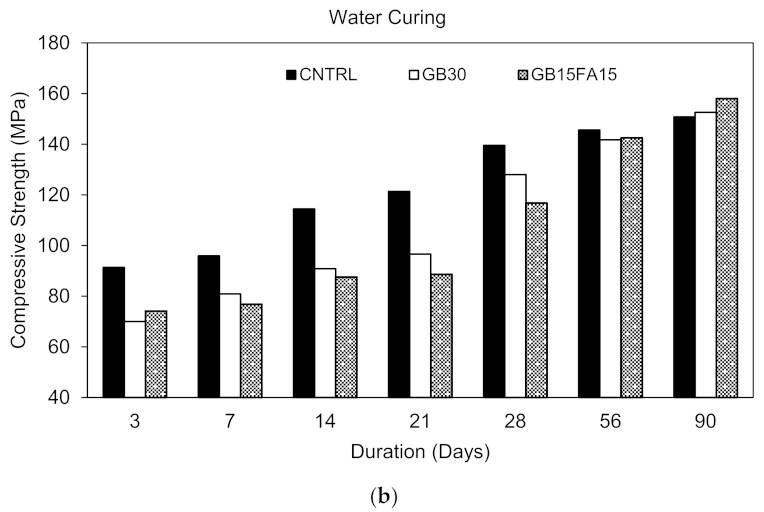
Compressive strength development. (**a**) HAC + ambient; (**b**) water curing.

The compressive strength results of the three mixes subjected to hot air curing and normal water curing are presented and discussed in this section. Figure 4 presents the 12 h increment strength development of HAC specimens, while Figure 5a,b present the 90-day strength development of 24-day HAC + ambient and WC specimens. All hot air cured samples were left in ambient condition 1 day post demolding to ensure that the concrete is dry before exposing to HAC. It is worth mentioning that when the samples were removed from the molds and put directly in the oven, this led to a significant drop in the compressive strength of RPC. When the samples were put directly in the oven for 24 h, the compressive strength did not exceed 115 MPa. However, when the samples were left an additional day in ambient condition prior to heat curing, the strength increased and ranged from 130 MPa to 155 MPa. This is probably because when the samples were removed from the molds, they were not completely dry. Exposing these samples directly to HAC probably affected the cement hydration and weakened the concrete microstructure due to the rapid vaporization of water. This consequently may have induced microcracks and pores within the concrete microstructure which therefore lowered the concrete strength as opposed to samples which were left an additional 24 h in ambient condition. Therefore, it is critical to monitor the time at which the samples are heat cured to avoid any adverse effect and to obtain the maximum strength enhancement.

#### 3.2.1. 48 h Hot Air Curing Strength Development

From Figure 4, it can clearly be seen that heat curing has shown significant effect on the early strength development of all mixes regardless of the constituents used. Before exposing the samples to heat curing at 0 h, the CNTRL mix had the highest strength of 77 MPa, followed by the GB30 and GB15FA15 mixes, which had strengths of 68 and 55 MPa, respectively. After exposing these samples to HAC, the strength significantly increased by 79%, 72%, and 137% for the 12 h curing, respectively, with the CNTRL mix yielding the highest 12 h strength of 138 MPa. After 12 h of heat curing, the strength development for the mixes became less evident.

As observed from Figure 4, the strength development of the control mix was different to that of the GB30 and GB15FA15 mixes. The strength of the control mix increased only until 24 h and then started to decline, whereas the strengths of the GB30 and GB15FA15 mixes continued to increase even after 24 h. This indicates that there is an optimal crystallization limit, which, if exceeded, lead to a decline in strength and unfavorable crystallinity. The highest strengths achieved overall were 155 MPa for 24 h HAC CNTRL mix, 140 MPa for 48 h HAC GB30 mix and 145 MPa for 48 h HAC GB15FA15 mix.

It is known from the literature that HAC significantly increases the strength of RPC [2,28]. In a study conducted by Hiremath and Yaragal [28], the specimens were exposed to HAC for 7 days, and the strength was observed to increase during the curing period. In the current investigation, however, the strength declined after 24 h of HAC for the control mix. This is because in [28], cement and SF were used alongside quartz, which is well known for its high silica content and reactivity under temperatures higher than 90 °C. This therefore might have prolonged the strength increase to up to 7 days. In this study, the GB30 and GB15FA15 mixes had higher silica content as compared to the control mix. This probably attributed to the continued strength increase observed in Figure 4 as opposed to the control mix with lower silica content. Although the strength of the GB30 and GB15FA15 continued to increase, the 24 h strength of the control mix was still higher than the 48 h mixes incorporating GGBS and FA due to the slow pozzolanic reactions of these materials as compared to cement. Yet, it is expected for these mixes to have comparable strengths to their control counterpart at longer durations of HAC.

#### 3.2.2. Hot Air Curing vs. Water Curing

All the hot air cured specimens were left in the oven for 24 h and then were left in ambient condition until 90 days. Although the highest strength for the GB30 and GB15FA15 mixes occurred at 48 h, the specimens were still removed from the oven at 24 h to avoid increased energy consumption and costs associated with heat curing.

As shown from Figure 5a, no specific trend was perceived for the compressive strength development of the control mix. The highest strength obtained was 160 MPa at 7 days, and the average strength reported was found to be 152 MPa. Conversely, the GB30 and GB15FA15 mixes exhibited different behavior and showed continued strength increase up to 90 days due to the incomplete hydration and delayed pozzolanic effect of the GGBS and FA. From Figure 4, it is clear that GGBS and FA mixes did not reach their ultimate strength at 24 h of HAC. This is probably why the strength increased up to 90 days for these mixes as shown in Figure 5a. The 90-day strength of the GB30 and GB15FA15 mixes was 155 and 156 MPa, respectively, with an approximate increase of 14% over the 90-day period of ambient curing.

Figure 5b shows the compressive strength of the water cured specimens from which it is observed that the strength continues to increase as the curing time increases. At 7 and 28 days of WC, the strengths of the specimens were around 56% and 92% of the 24 h HAC. At 90 days of WC, the strength of the CNTRL mix was around 100% of the 24 h HAC, indicating that the 90-day WC strength of the control mix can be achieved within 24 h only under HAC. In contrast, the mixes incorporating GGBS and FA at 90 days of WC had slightly higher strength than that of the 24 h HAC due to the incomplete hydration at 24 h HAC. However, under HAC (Figure 4) when the strength further increased, the strengths of the 90-day WC specimens were similar to those of the 48 h HAC. Although the 90-day WC strength of the GGBS and FA mixes was slightly higher than the 24 h HAC specimens, when these mixes were left in ambient condition until 90 days, the strengths were comparable to those of the 90-day WC and 48 h HAC specimens.

#### 3.2.3. Effect of GGBS and Ternary Blends

The incorporation of 30% GGBS reduced the strength at early ages under all curing conditions with a strength decrease not exceeding 20%. These results are in line with [5,10,29] as the use of GGBS is known to reduce the strength at early ages. At HAC + ambient and WC, the strength of the GB30 mix increased and became analogous to that of the control mix, as shown Figure 5a,b. The GB15FA15 mix exhibited similar behavior to that of the GB30 mix as opposed to their counterpart mix. Moreover, when comparing the effect of the ternary system SF-GGBS-FA versus the binary system SF-GGBS, no major difference was detected. In fact, both mixes exhibited similar behavior with trivial variations. Although it was shown in the literature that ternary blends enhance the strength properties as compared to binary blends [16,19], this was not observed in this study. The results of this study are in agreement with [5], which showed that ternary blends of SF-GGBS-FA only showed better performance than binary blends of SF-FA and not SF-GGBS.

### 3.3. Flexural Strength

**Figure 6:**
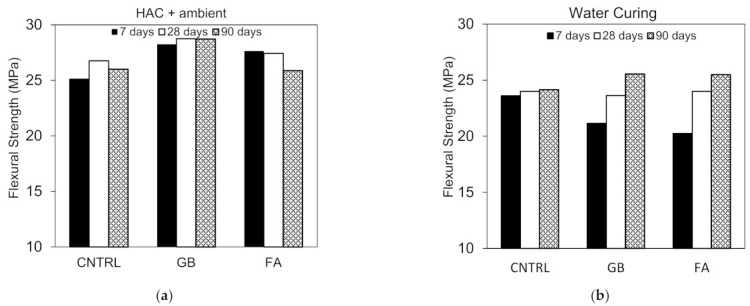
Flexural strength development. (**a**) HAC + ambient; (**b**) water curing.

The HAC and WC flexural strength results are shown in Figure 6a,b. Overall, the results show that the contribution of the HAC to the strength was more apparent for the compressive strength as opposed to the flexural strength. These findings are in line with the majority of the studies in the literature which studied the effect of thermal and hydrothermal curing regimes [12,30,31]. The results of Figure 6a,b show that the strength of hot air cured specimens was higher than that of the water cured specimens for all mixes with an increase ranging mostly from 6% to 14%. There are several plausible reasons which might have contributed to the strength increase and these include the increased reactivity of silica components and the reduced porosity which occur as a result of the enhanced rate of C-S-H production. Additionally, since the coefficient of concrete shrinkage is close to that of the thermal expansion, it is likely that the increase in temperature might have reduced the internal strains resulting from shrinkage, which in turn might have contributed to the strength increase [31].

Hot air curing in general is not studied to the same extent as steam curing and autoclave curing in the literature. A study conducted by Xun et al. [31] showed that the increase in flexural strength did not exceed 13% after exposing the samples to hot air curing. Despite the results obtained in the current investigation, it is worth mentioning that in the literature different trends were observed for the flexural strength development under thermal and hydrothermal curing regimes. For example, in a series of studies conducted by Yazici et al. and Aydin et al., the flexural strength decreased under steam curing with a maximum decrease reaching up to 18% [7,32,33]. For autoclaving, on the other hand, no major strength increase was reported for the flexural specimens. In fact, some specimens showed reduced flexural strength [32]. In these studies, the decrease in strength was mainly ascribed to the formation of a weaker bond between the fiber-and-matrix bond under such curing regimes. Conversely, in a study conducted by Zdeb [30] it was clearly shown that the flexural strength significantly increased when either steam or autoclave curing were applied, and the strength increase reached beyond 50%. Despite these contradicting results, the majority of the studies in the literature, including the results of the present investigation, showed that the flexural strength improvement is limited as compared to the compressive strength and mostly does not exceed 30%. One possible justification could be due to the increased porosity which occurs as a result of high temperature, thereby weakening the fiber-and-matrix bond and limiting the strength improvement. Note that the microstructure of HAC specimens can be seen in the following section in the SEM investigation.

The inclusion of GGBS increased the flexural strength by up to 12% for the hot air cured specimens. For the water cured specimens, the strength decreased (10%) at 7 and 28 days and increased at the later age of 90 days by 5%. This improvement in strength can be attributed to the improvement in bond strength over time with the use of GGBS. Similar to the compressive strength behavior, the use of ternary blends of SF-GGBS-FA did not show any major difference for the flexural strength compared to the binary blends of SF-GGBS. Both mixes showed comparable strength under water curing while the GB30 mix showed slightly higher strength (2.16–10%) under HAC.

### 3.4. Microstructure Investigation

Microstructure of the selected RPC specimens was studied using a scanning electron microscope (SEM), equipped with a secondary detector and energy dispersive X-ray (EDX) analysis system. The effect of different curing conditions on the RPC microstructure and the fiber-to-matrix bond are discussed in this subsection.

#### 3.4.1. Effect of Different Curing Conditions

**Figure 7:**
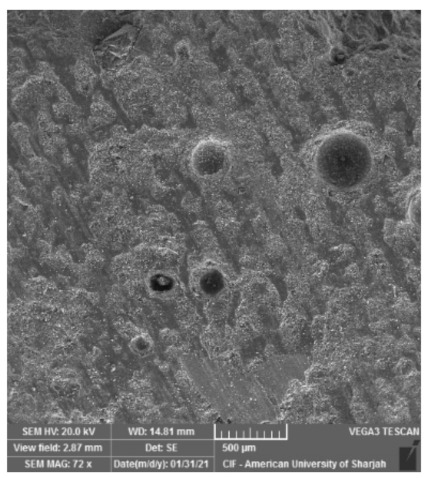
Entrained and entrapped air voids in RPC microstructure.

**Figure 8:**
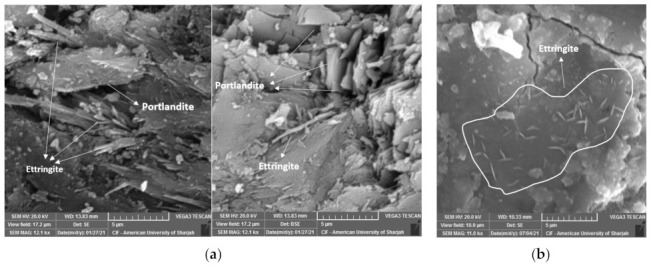
RPC microstructure under water curing. (**a**) early-age ettringite and portlandite; (**b**) late-age ettringite.

Microstructure investigation demonstrates the dense and homogeneous structure of RPC. Entrained and entrapped air pores were found in all mixes possibly as a side effect of the high amount of superplasticizer (Figure 7). In general, when C_3_S and C_2_S react with water, they are transformed into C-S-H and hydrated lime (Ca(OH)_2_). The chemical composition and morphology of the C-S-H vary, which is why it is referred to vaguely as C-S-H. C-S-H compromises about 60–70% of fully hydrated cement and is the main source of strength development in concrete. Portlandite on the other hand is the crystalline form of the hydrated lime. At 3 days of water curing, the formation of strength reducing ettringite (C_6_AS_3_H_32_) was detected in the control mix, as shown in Figure 8a. These compounds are small needle-bar shaped structures and hinder the rate of hydration, which in turn slows the strength development under water curing. Portlandite crystals can also be seen in Figure 8a and tend to have a marginal effect on the mechanical properties of concrete [34]. With the increase in curing period, compounds such as ettringite tend to get smaller with time (Figure 8b) until they eventually disappear.

**Figure 9:**
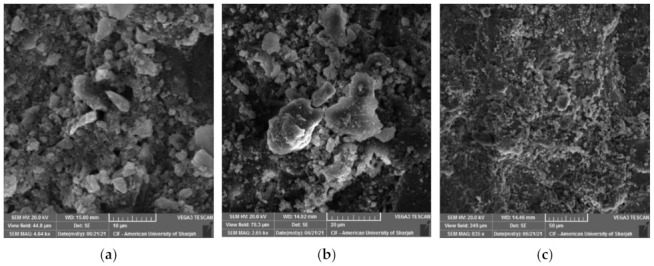
RPC microstructure HAC. (**a**) 12 h; (**b**) 24 h; (**c**) 48 h.

While looking at the microstructure of the HAC specimens, ettringite was not detected even at 12 h of HAC (Figure 9a). Portlandites react with pozzolanic materials to form additional C-S-H. At high temperatures, the rate at which these reactions occur increases and this eventually leads to higher early strength development as observed for the 12 h HAC specimens. However, it is important to note that in the absence of silica sources, heat curing regimes are not desired. This is because other compounds such as truscottite or alpha dicalcium silicate hydrates (α-C_2_SH) form and weaken the aggregate- and fiber-to-matrix bond [35]. In the presence of silica sources, α-C_2_SH converts to other desirable forms of C-S-H products such as tobermorite and xonotlite. The presence of tobermorite-like structures was previously reported by many studies under autoclaving curing conditions.

**Figure 10:**
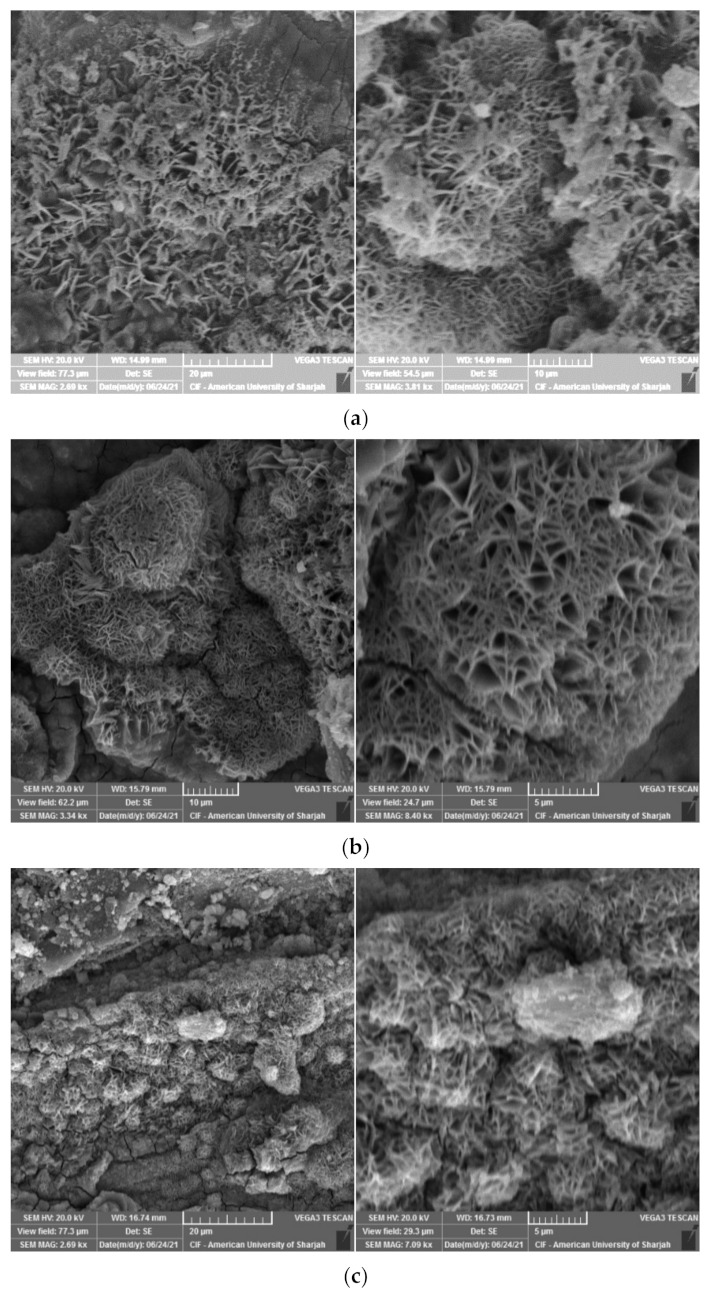
Iron oxide products spotted at 90 days of 24 h HAC specimens. (**a**) CNTRL mix; (**b**) GB30 mix; (**c**) GB15FA15 mix.

**Figure 11:**
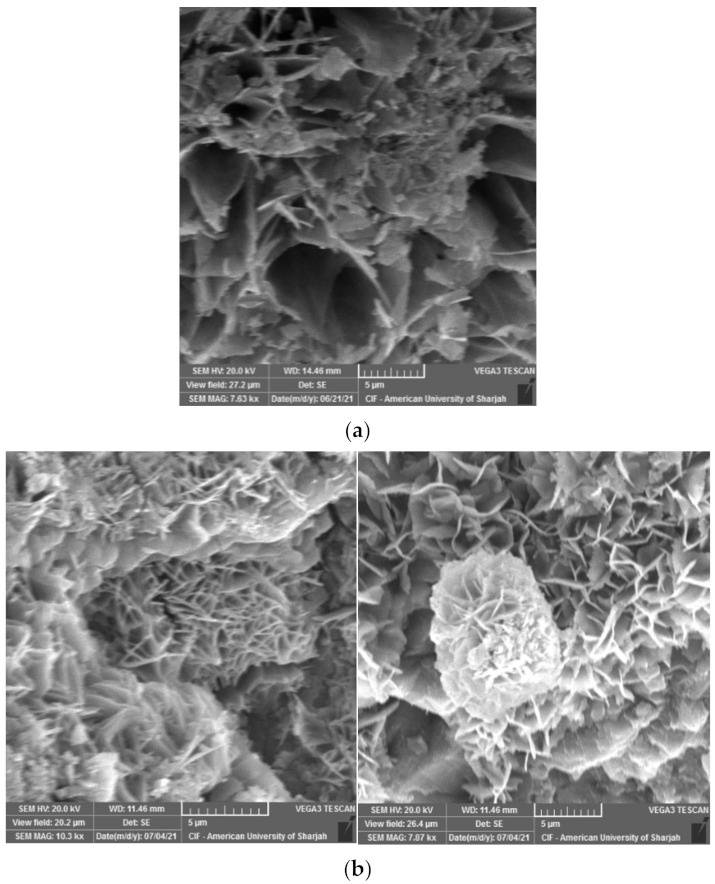
Iron oxide products for the GB15FA15 mix. (**a**) early age HAC; (**b**) late age WC.

In this study, tobermorite was not detected for the 24 h and 48 h HAC specimens as shown in Figure 9. However, at late ages, when these samples were left in ambient condition, foiled plate-like structures formed (Figure 10), which visually appeared to be similar to the tobermorite structures reported in [5,29,36]. Particularly, they were mainly observed for HAC specimens for all the mixes. When EDX was conducted to identify the elemental composition of the material observed, results showed high concentrations of Fe and O with the absence of silica, indicating that the compounds detected are not tobermorite and rather are corrosion iron oxide products. The morphologies observed are similar to those reported by several authors [37,38,39] and may be characterized as goethite and hematite. Nevertheless, it should be emphasized that these compounds mainly appeared for HAC specimens at the age of 90 days. In addition, a few of these products were detected at early ages of HAC and 90 days of WC for the mix incorporating fly ash only (Figure 11). This denotes that the use of FA can accelerate the formation of these compounds. In general, corrosion products initiate at the surface of the steel and then expand up to several times of their initial size. This induces pressure which can lead to cracking and spalling of the concrete. However, this process initially causes an increase in the bond due to the increased toughness which is later reduced with further growth in corrosion [40]. From a mechanical point of the view, the compressive strength (Figure 5a) was not clearly affected by the formation of these products, as the highest strength for the GGBS and FA mixes was reported at 90 days for both WC and HAC specimens. On the contrary, the flexural strength results (Figure 6a) showed that the strength of the control and GB15FA15 mixes decreased at 90 days. This may imply that the formation of these products weakened the bond strength between the steel fibers and the matrix, which in turn may have reduced the strength of the flexural specimens at 90 days. From the results of the present study, it is critical to study the durability of steel fiber-reinforced RPC over the long term to further confirm the effect and rate of formation of these compounds in RPC subjected to thermal curing conditions.

#### 3.4.2. Steel-to-Matrix Bond

**Figure 12:**
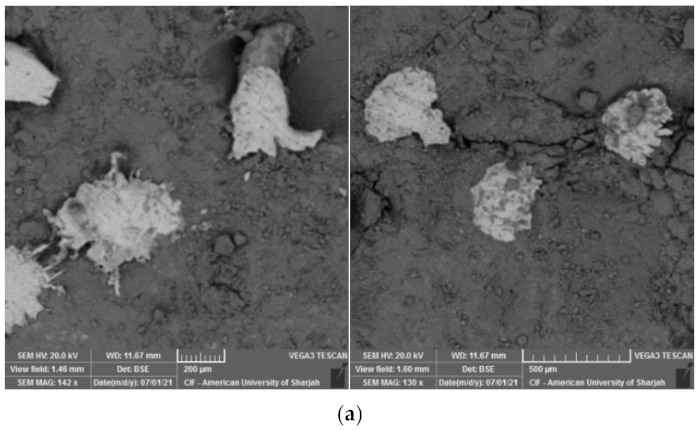

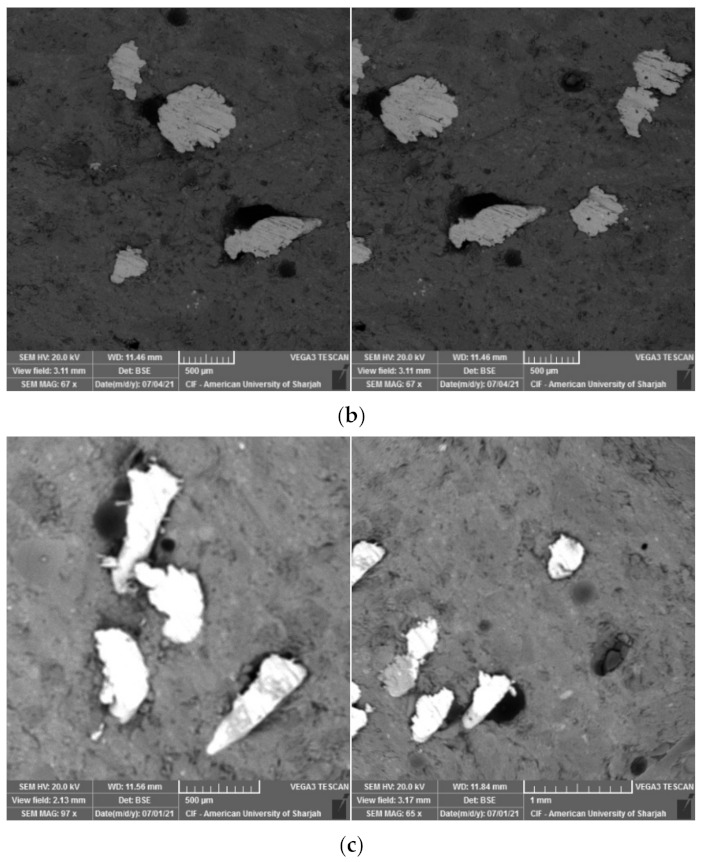
Fiber-matrix bond under early ages of WC. (**a**) CNTRL mix; (**b**) GB30 mix; (**c**) GB15FA15 mix.

The steel fiber-to-matrix bond was investigated for the mixes evaluated in this study. At early ages of water curing, clear interfacial transition zones (ITZ) can be detected, as demonstrated in Figure 12. Microcracks are depicted in the microstructure of these mixes. The control mix seems to have better bonding as compared to the GGBS and FA mixes. Clear voids are observed at the steel fiber-to-matrix bond of the GB30 and GB15FA15 mixes. This probably occurred as a side effect of the slow rate of reaction of GGBS and FA which further contributed to the early age low strength.

**Figure 13:**
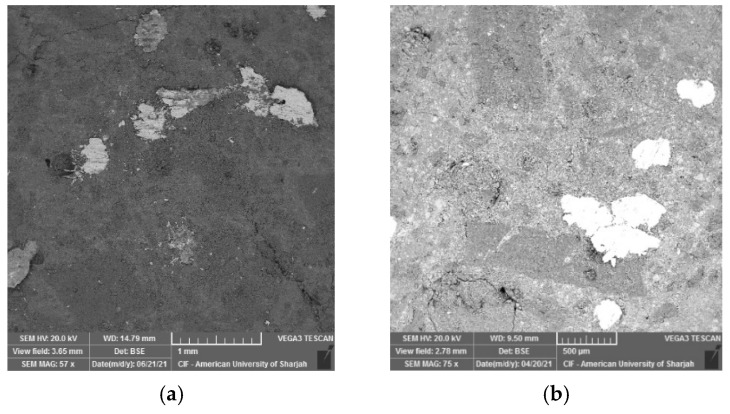
Fiber-matrix bond at late ages of HAC. (**a**) GB30 mix; (**b**) GB15FA15 mix.

**Figure 14:**
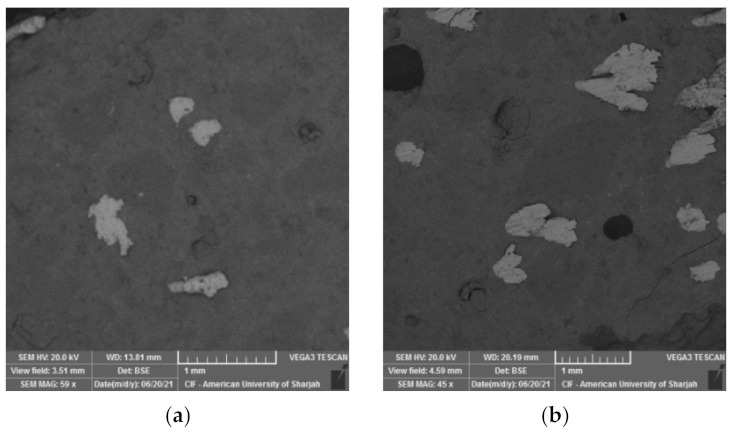
Fiber-matrix bond at late ages of WC. (**a**) GB30 mix; (**b**) GB15FA15 mix.

The steel fiber-to-matrix bond is shown for the late age of WC and HAC in Figure 13 and Figure 14, respectively. It is obvious that all mixes at this stage show a denser microstructure with fewer voids and microcracks. In addition, the fibers are well dispersed in the matrix without any clear agglomeration. This is due to the consumption of Ca(OH)_2_ crystals with HAC and long-term water curing which eventually led to the formation of a denser matrix with a stronger fiber-to-matrix bond. From Figure 13 and Figure 14, it can also be observed that WC tends to have a denser microstructure compared to HAC at late ages. This is because high temperature causes rapid hydration coupled with low moisture content, which results in C-S-H with a more porous microstructure, unlike WC, which provides a continuous moisture supply [41].

## 4. Conclusions

This paper was an attempt to produce reactive powder concrete using indigenous materials. The key findings of the present investigation are concluded as follows:

- HAC significantly increased the compressive strength of all mixes as compared to the flexural strength. Up to a 137% percentage increase was reported for the 12 h HAC, while for the flexural strength the percentage increase only reached up to 37% and mostly ranged from 6% to 14% for the 24 h HAC.
- The use of GGBS and FA is more desirable under heat curing conditions as the compressive strength of GB30 and GB15FA15 continued to increase under HAC as opposed to the control mix.
- The 90-day WC strength of the CNTRL mix can be achieved within 24 h only of HAC, while for the GB30 and GB15FA15, higher HAC durations are required to achieve the 90-day WC strength. However, it should be noted that when the specimens of the GGBS and FA mixes were heat cured for 24 h only and left in ambient condition until 90 days, the strengths were comparable to that of the 90-day WC specimens.
- The time at which the specimens are exposed to heat curing has a major impact on the properties of RPC. When the specimens were demolded at 24 h and hot air cured, the compressive strength did not exceed 115 MPa. However, when the samples were left an additional day in ambient condition and then subjected to HAC, the strength exceeded 120 MPa after 12 h only. Therefore, it is critical to monitor the time at which the concrete specimens are subjected to HAC to avoid any adverse effect on the RPC performance.
- Microstructure investigations revealed the formation of iron oxide (corrosion) products for all HAC mixes at late ages. The incorporation of FA increased the rate at which these products formed as they were detected at early ages of HAC and late ages of WC for the GB15FA15 mix. This suggests that the use of GGBS is more durable as compared to FA. However, the effect of the formation of these products at late ages should still be studied from a durability point of view. In addition, additional research is required to study the duration at which these compounds form in concrete.
- Finally, it can be concluded from the experimental work results that the optimum mix of this study is the GB30 mix, and the production of ultra-high strength RPC is possible with the use of crushed and dune sand with aggregate sizes of 150 µm–1.18 mm.

## Figures and Tables

**Table 1:** Chemical composition and physical properties of different binder materials.

	Cement	SF	GGBS	FA
SiO_2_ (%)	20.5	92.98	-	64.50
Fe_2_O_3_ (%)	3.6	1.49	-	-
Al_2_O_3_ (%)	4.8	0.49	-	-
CaO (%)	63	-	-	-
MgO (%)	2.3	0.57	6.0	0.39
K_2_O (%)	-	0.51	-	-
Na_2_O (%)	0.54	0.47	-	0.21
SO_3_ (%)	2.6	0.57	0.10	0.047
LOI	-	1.80	-	0.92
Blaine fineness (m^2^/kg)	318	16,000	417	474.2
Initial setting time (min)	180	-	-	-
Final setting time (min)	230	-	-	-
7-day compressive strength	40.2	-	40.6	-
28-day compressive strength	52.6	-	54.8	-

**Table 2:** Mix proportions.

	CNTRL	GB30	GB15FA15
Cement (kg/m^3^)	818	571	571
SF (kg/m^3^)	272	272	272
GGBS (kg/m^3^)	-	222	122
FA (kg/m^3^)	-	-	122
Crushed sand (kg/m^3^)	633	633	633
Dune sand (kg/m^3^)	458	458	458
Water (kg/m^3^)	185	185	185
Steel fibers (kg/m^3^)	141	141	141
Water-to-binder ratio	0.17	0.17	0.17
Aggregate-to-binder ratio	1.0	1.0	1.0

**Table 3:** Summary of mechanical tests conducted.

Mechanical Tests	Standard	Specimen Size	No. of Test Specimens	Curing Condition		
				WC	HAC	HAC + Ambient
Compressive strength	ASTM C 109	50 × 50 × 50 mm	4	3, 7, 14, 21, 28, and 90 days	12, 24, 36, and 48 h	3, 7, 14, 21, 28, and 90 days
Flexural strength	ASTM C 293	40 × 40 × 160 mm	3	7, 28, and 90 days		7, 28, and 90 days

## Data Availability

The data presented in this study are available on request from the corresponding author.
